# Exploring the Developmental Progression of Endosperm Cavity Formation in Maize Grain and the Underlying Molecular Basis Using X-Ray Tomography and Genome Wide Association Study

**DOI:** 10.3389/fpls.2022.847884

**Published:** 2022-04-07

**Authors:** Shengjin Liao, Ying Zhang, Jinglu Wang, Chunjiang Zhao, Yong-Ling Ruan, Xinyu Guo

**Affiliations:** ^1^Beijing Key Laboratory of Digital Plant, Research Center of Information Technology, Beijing Academy of Agriculture and Forestry Sciences, Beijing, China; ^2^National Watermelon and Melon Improvement Center, Beijing Academy of Agriculture and Forestry Sciences, Key Laboratory of Biology and Genetic Improvement of Horticultural Crops (North China), Beijing Key Laboratory of Vegetable Germplasm Improvement, Beijing, China; ^3^Research School of Biology, The Australian National University, Canberra, ACT, Australia

**Keywords:** maize, endosperm cavity, X-ray micro-computed tomography, GWAS, development

## Abstract

Endosperm cavity (EC) in maize grain reduces yield and causes grain breakage during mechanical harvesting, hence representing a major problem in the maize industry. Despite this, little is known regarding the biological processes governing EC formation. Here, we attempted to address this issue by (i) determining the spatial and temporal progression of EC in a non-invasive manner and (ii) identifying candidate genes that may be linked to the formation of EC by using a genome wide association study (GWAS). Visualization and measurement using X-ray micro-computed tomography established that EC first appeared at the central starch endosperm at about 12 days after pollination (DAP) and became enlarged thereafter. GWAS-based screening of a panel of 299 inbred lines with a wide range of EC size identified nine candidate genes that showed significant association with EC formation. Most of the candidate genes exhibited a decrease at 12 DAP, coinciding with the timing of EC appearance. Among them, *ZmMrp11* was annotated as a member encoding a multidrug resistance-associated protein that has been shown in other studies to sequestrate toxic metabolites from the cytosol to the vacuole, thereby detoxifying the cellular environment. This, together with the reduced expression of *ZmMrp11* in maize grains from 12 DAP, prompted us to propose that the low expression of *ZmMrp*11 may block cellular detoxification in the maize endosperm cells, leading to cell death and ultimately the formation of EC.

## Introduction

The inner structure of a given organ is of fundamental importance in determining its biological function and chemical and physical properties. Visualization of cellular structures deeply embedded inside an organ often requires the time-consuming and labor-intensive preparation of sections followed by microscopic observation. Moreover, these methods are usually destructive and lack the capability for quantification of specific traits, such as cellular volume.

In this context, X-ray micro-computed tomography (μCT) scanning technology, widely used in medical imaging, has emerged as an ideal tool to non-invasively visualize and quantify the inner structures of plant organs (Schoeman et al., 2016). For example, it has been applied to estimate the hardness of maize grains by calculating the porosity of the grain (Guelpa et al., 2015) and to explore the relationship between maize grain geometry and grain breakage susceptibility (Hou et al., 2019). The X-ray μCT has also been employed to track the changes in the internal structure of the maize kernels followed by infection with *Fusarium verticillioides* (Orina et al., 2017) and to examine the endosperm organization of high-amylose rice grains (Zhu et al., 2012), the gap between maternal and filial tissues of wheat grain as well as the effect of endosperm microstructure on the cooking behavior of rice grain (Mohorič et al., 2009).

Maize (*Zea mays* L.) is a major staple crop worldwide. It partitions about half of its above-ground dry mass to the ears that develop the largest grains among cereals (DeLucia et al., 2019). The bulk of the mature maize grain is the endosperm, which constitutes 80–90% of the mature kernel’s dry weight (Kowles and Phillips, 1988). To this end, the formation of endosperm cavity (EC) during development results in a significant reduction of maize grain yield (Deng et al., 2020) and grain hardness (Guelpa et al., 2015). The latter leads to grain breakage during mechanical harvest and consequently loss of profitability (Guelpa et al., 2015). A similar problem also exists in other organs such as wheat grains (Grimberg et al., 2020).

Despite the negative impact of EC on maize grain yield and quality, and hence on the overall success of the maize industry, little is known regarding the biological process governing EC formation, namely, when, where, and how ECs are formed during grain development. As the first step to address this issue, we employed the X-ray μCT technology to locate and quantify the EC formed within the maize kernel during development. We then attempted to identify genes that may involve in EC formation by analyzing a population consisting of 299 inbred maize lines with a wide range of EC sizes using a genome wide association study (GWAS).

## Materials and Methods

### Plants Material and Growth Condition

A panel of 299 maize inbred lines, as reported by Yang et al. (2011), was planted in the Sanya Experimental Station of Beijing Academy of Agriculture and Foresting Sciences in Hainan, China in 2018. Irrigation, fertilization, and pest or disease control were performed according to Zhang et al. (2021).

### Preparation of Maize Kernels for X-Ray Tomography

The young developing kernels were dehydrated before X-ray tomography analyses. The dehydration processes were conducted according to Zhang et al. (2021) with slight modifications. Briefly, young kernels were harvested and immersed in formaldehyde–acetic acid fixative containing 50% ethanol, 5% glacial acetic acid, and 3.7% (v/v) formaldehyde immediately. After fixation, the samples were dehydrated through a sequential ethanol series from 50, 70, and 95–100% at a 24-h interval for each gradient. Then, samples were then transferred to tertiary butyl alcohol for 24 h before being frozen at −80°C overnight. Thereafter, the frozen kernels were freeze-dried in a freezer dryer machine (LGJ-10E, China). The mature and naturally dried kernels were scanned directly by X-ray μCT without the drying process. Three natural dried mature kernels harvested from the middle part, 1/3 of ear length in the ear central (Shen et al., 2018), of a representative ear were collected from each inbred line for phenotypical analysis.

### Non-invasive Detection and Measurement of Cavities Within Maize Kernels Using X-Ray Tomography

A Skyscan 1172 X-ray μCT system (Bruker Corporation, Billerica, MA, United States) was used to acquire spatial information on the inner cavity of maize kernels of the 299 inbred lines with a unified setting, as described by Hou et al. (2019). Briefly, kernels were scanned with the imaging pixel of 13.5 μm in the 2K scanning mode (2000 × 2000 pixels). The sample was scanned over 180° rotation with an image taken at every 0.2°. For each sample, the X-ray μCT scan generates about 900 images. To further quantify the size (volume) of the kernel’s inner cavity, a series of 2D X-ray images was converted into a 3D image using the X-ray system integrated software Nrecon, which transferred the gray level of the raw image (16 gray level) into an 8-bit gray-level image ranging from 0 to 255, corresponding to white and black, respectively.

The principle of X-ray μCT analyses is based on the differences in X-ray attenuation arising from differences in material density and composition (Chawanji et al., 2012). The average attenuation of the sample is expressed in Hounsfield Unit (HU) or CT number (Donis-González et al., 2014). Thus, the differences in physical density or constituents are observed as changes in the HU or CT number. For example, the air has a low HU of −1,000, whereas a solid material with high density may have a HU value up to 3,000 (Donis-González et al., 2014). In the X-ray μCT image, the attenuation variance of different structural components within a sample was indicated by gray-level intensities, where the denser the region, the higher the attenuation, and the brighter the region appears on the image.

X-ray μCT analyses of grains from the maize inbred line revealed that the gray level of the cavity region in the grain was less than 50. Hence, the gray intensity of the X-ray image that was less than 50 was used as a threshold to calculate the EC size of the kernel. In this study, the batch function of CTAn software associated with the X-ray μCT system was employed for the high-throughput calculation of the cavity size for the 299 inbred lines. A 3D image processing software, ScanIP (Simpleware Ltd., Exeter, United Kingdom), was used to measure the volume of each cavity in maize grains as described by Hou et al. (2019).

### Genome-Wide Association Analysis

The GWAS was performed as described by Lu et al. (2021) and Zhang et al. (2021) with minor modifications. Briefly, genotype data of the 299 inbred lines were obtained from Professor Yan Jianbing’s laboratory of Huazhong Agricultural University (URL^1^). A total of 779,855 SNPs with a minimum allele frequency (MAF) greater than 0.05 and a call rate greater than 0.9 were used for GWAS analysis (Biscarini et al., 2016). A multi-locus random-SNP-effect mixed linear model tool (R package “mrMLM” version 4.0) coupled with the population structure (Q) and kinship (K) data was used to test the statistical association between the cavity trait and the genotypes. Six multi-locus GWAS methods (mrMLM, FASTmrMLM, FASTmrEMMA, ISIS EM-BLASSO, pLARmEB, and pKWmEB) were included in the “mrMLM” analyses. These six ML-GWAS methods were processed in two steps. First, each SNP on the genome was filtered with a *p* ≤ 0.5/N, where *N* is the total number of genome-wide SNPs. Then, all the SNPs that were potentially associated with the trait were included in a multi-locus genetic model further screened with a defeat *p*-value of 0.0002 to declare a significance of SNPs that were associated with a given trait. Only SNPs that were identified by more than two multi-locus GWAS methods were regarded as ‘‘Top’’ SNP. Annotation of SNPs was conducted by using ANNOVAR software against the maize B73 reference genome (B73 RefGen_v4) available in Ensembl Plants^2^ and NCBI Gene database^3^.

### Histological Analysis of Developing Kernels

The histological section of developing kernels staged at 8, 10, 12, and 14 DAP were prepared according to Zhao et al. (2018) with minor modification. The kernels were harvested freshly from the middle of the ear and immersed immediately in the formaldehyde–acetic acid fixative containing 50% (v/v) ethanol, 5% glacial acetic acid, and 3.7% (v/v) formaldehyde at 4°C overnight. Then, the samples were dehydrated through a gradient of ethanol, xylene, and embedded in paraffin. Samples were sectioned with a microtome at 8 μm in thickness (Leica Microsystems, Wetzler, Germany), stained with toluidine blue O (TBO), and pictured by Zeiss microscopy (Jean, Germany) with a CCD Camera.

### Statistical Analyses

JMP14 software was used in this study for data analysis. Detailed methods are captioned in the figure legend.

## Results

### Determining the Spatial and Temporal Formation of Endosperm Cavity

**FIGURE 1:**
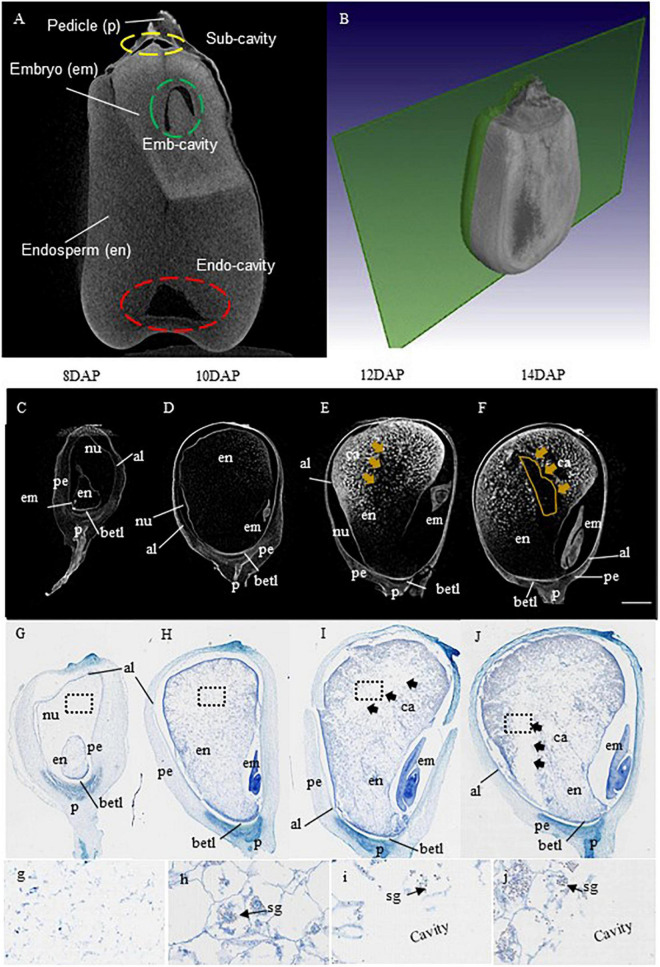
X-ray μCT and histological demonstrations on the temporal and spatial formation of maize endosperm cavity. **(A,B)** Representative sagittal views of a mature grain of maize B73 with an X-ray μCT image on the left and the corresponding schematic 3D sectional view on the right quoted from Schoeman et al., 2016. Based on the difference in X-ray attenuation of different constituents, different components of the kernel were distinguished by gray level intensities, with brightest and the darkest gray regions representing the embryo, and the cavities, respectively. Note the endosperm exhibited gray intensity was lighter than that of the embryo but stronger than the cavities. The cavities were highlighted with yellow, green, and red-dashed circles for subcutaneous cavities (Sub-cavity), embryo cavity (Emb-cavity), and endosperm cavity (Endo-cavity), respectively. **(C–F)** Progression of endo-cavity formation revealed by X-ray μCT analysis on B73 maize kernels at stages 8, 10, 12, and 14 day after pollination (DAP). The cavity first appeared in the central starch endosperm region at 12 DAP and became evident at 14 DAP, shown as dark regions of the X-ray images, marked by yellow arrow **(E,F)**. Bar = 1 mm. **(G–J)** Longitudinal section of B73 kernel at stage 8, 10, 12, and 14 DAP stained with toluidine blue. The images of g-i corresponded to the magnified view of the black dash boxed regions in **(G–J)** at 8, 10, 12, and 14 DAP, respectively, showing the progression of the morphological formation of endo-cavity. The cavity was visible at 12 DAP and became apparent at 14 DAP, denoted by black arrows **(I,J)**. Note, starch granules were observed in the cellularized endosperm cells (arrows) but not in the cavities as expected (I,j). al, aleurone layer; em, embryo; en, endosperm; nu, nucellus; per, pericarp, ca, cavity; betl, base endosperm transfer layer; p, pedicle; sg, starch granule. Bar = 1.25 mm.

To investigate when and where the EC is formed, we performed a detailed X-ray μCT analysis on maize kernels starting from 8 DAP at 2-day intervals. Figure 1A represents a sagittal view of mature maize grain with the corresponding schematic 3D sectional view on the right (Figure 1B) from B73, a standard maize line from which the reference genome was produced (B73 RefGen_v4). Different tissues of the kernel were distinguished by different levels of the gray intensities, with the brightest and the darkest gray regions representing the embryo and the cavities, respectively (Figure 1A). Here, the embryo area represents a high level of attenuation indicating dense structure, while the cavities had the lowest attenuation, thus showing the darkest area.

As shown from the X-ray μCT imaging, the grain cavities consist of EC, embryo cavity, (EmC), and subcutaneous cavity (SubC, the gap between the pericarp and the endosperm), with EC being the largest among the three (Figure 1). It is worth noting that the EC described here is different from that of the EC in wheat. The latter is designated as a gap between the endosperm and the maternal tissue (Chateigner-Boutin et al., 2021) from which nutrients are taken up by the filial tissues. By contrast, the EC in maize grain represents a hollow space within the basal region of the endosperm (Figure 1A).

We then examined the temporal progression of EC formation by conducting X-ray μCT analyses on B73 maize kernels at stages 8, 10, 12, and 14 DAP. In the maize grain, the cellularized endosperm starts to accumulate starch from 8 DAP onward (Zhan et al., 2015). Consistent with this physiological feature is the increase in gray intensity in the endosperm from 8 to 14 DAP, reflecting the increasing accumulation of storage products and tissue density (Figures 1C–F). The overall dark appearance of the grain filial tissue at 8 and 10 DAP (Figures 1C,D) indicates low structural density, reflecting the low level of endosperm starch accumulation at these stages.

Surprisingly, in contrast to the overall increase of gray intensity in the endosperm as starch accumulates, a dark hollow space (cavity) appeared at 12 DAP in the upper part of the starch endosperm (Figure 1E), which enlarged further in the 14 DAP kernel (Figure 1F). To further confirm the timing and location of the emergence of this EC, the same set of maize kernels used for X-ray scanning was sectioned and stained with toluidine blue (Figures 1G–I). The histological analyses displayed an overall increase in endosperm cellular density from 8 to 14 DAP (Figures 1G–J). Most notably, the analyses revealed the EC was formed in the endosperm at 12 DAP and further expanded at the 14 DAP (Figures 1I,J). These observations are in agreement with that from the X-ray μCT analyses (Figures 1C–F). A magnified view of the indicated regions revealed the accumulation of starch granules in the endosperm cells at 10, 12, and 14 DPA but their absence at 8 DAP and the EC regions at 12–14 DAP (Figures 1G–J). Collectively, the X-ray μCT and histological analyses established that the EC was formed in the central starch endosperm at 12 DAP and enlarged at 14 DAP.

### Establishing the Correlation Between Endosperm Cavity Size and Total Grain Cavity Volume for High Throughput Calculation

While the X-ray μCT system provides a solution to visualize the inner structure of the maize grain by applying a gray-level threshold (less than 50) to discriminate the cavity region from the surrounding non-cavity region, which enables the quantification of the total cavity size within a grain, it cannot specifically measure the volume of individual cavities such as EC due to the computing algorithm setting in the associated CTAn software. By incorporating the ScanIP software, we have recently developed an X-ray μCT-based micro-phenotype analysis process, which enables the measurement of individual cavities, although still a time-consuming process (Zhao et al., 2021). By analyzing grains from 11 different varieties differing in EC sizes revealed that EC is the predominant cavity in the maize kernel, which accounts for over 50% of the total cavity in the majority of the varieties (Zhao et al., 2021). This observation, together with the finding that the hardness of maize kernel is negatively correlated with inner cavity sizes (Guelpa et al., 2015), strongly indicates that the presence of EC reduces the hardness of maize kernel, thereby increasing its vulnerability to breakage.

**FIGURE 2:**
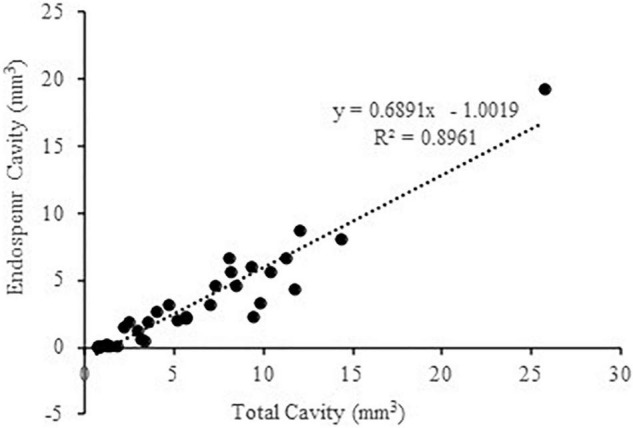
Correlation analysis between the endosperm cavity size and total cavity size in main grains. The analyses were performed on original data sourced from Zhao et al. (2021) for the 11 maize varieties differing in the size of endosperm cavity and total cavity.

We then performed a correlation analysis among these 11 varieties, which showed that the EC size closely correlated with the total cavity size of the entire kernel (sum of Ecs, EmC, and SubC), with *R*^2^ = 0.8961 (Figure 2). Given that it is widely accepted in genetic studies on crops including maize to apply a description factor highly correlated with a target phenotype as a proxy for GWAS (e.g., Mantilla-Perez et al., 2020), we next used the total cavity volume of the entire kernel as a proxy for the high throughput calculation of EC size for the GWAS analyses as described below.

### Identifying Loci and Candidate Genes Linked to Endosperm Cavity Formation by Performing Genome Wide Association Study

**FIGURE 3:**
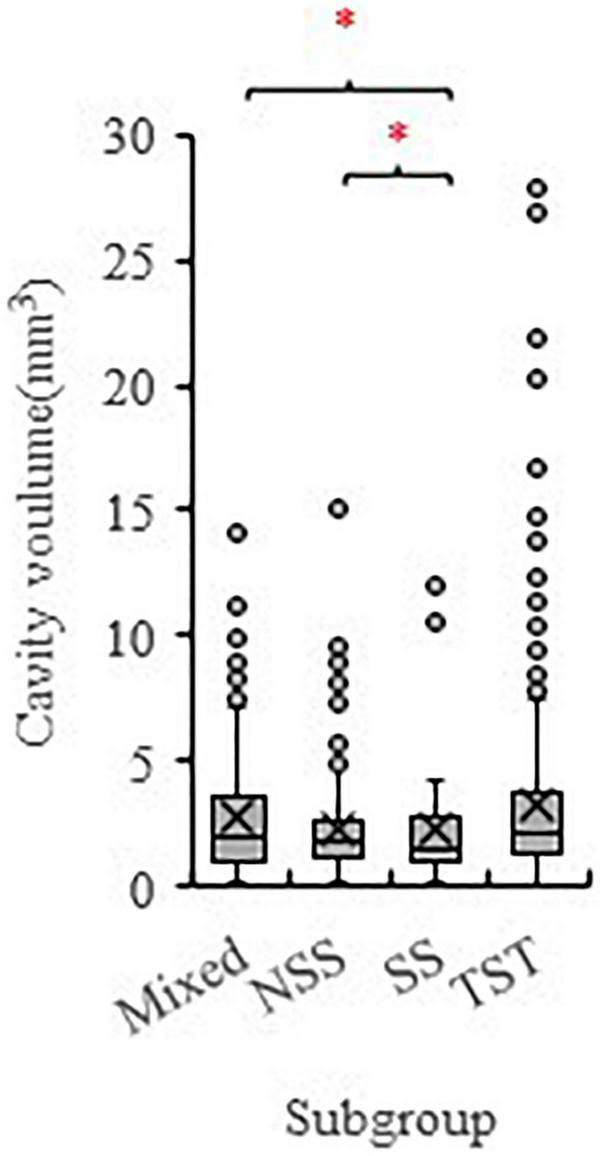
The phenotype variation of the inner cavity volume of maize kernels among different subpopulations (TST, NSS, SS, and Mixed). The 299 maize inbred lines association population consists of 131 TST lines, 81 NSS lines, 14 SS lines, and 73 Mixed lines. SS, Stiff stalk; NSS, non-stiff stalk; TST, tropical-subtropical. * symbolizes significant difference at *P* < 0.05 (Student’s *t* test).

To identify potential loci and candidate genes linked to the development of EC, we used the data of total inner cavity size from the 299 inbred maize lines (Supplementary Table 1) as a proxy for the EC trait to perform GWAS. According to the population structure analysis conducted by Yang et al. (2011), the 299 maize panel was classified into four subgroups with 131 lines in the tropical–subtropical (TST) group, 14 in Stiff stalk (SS), 81 in the non-stiff stalk (NSS), and 73 in a mixed subpopulation. Overall, the inner cavity size of the maize kernel showed a wide variation in the association panel. The inner cavity size of the TST subgroup was significantly higher than the SS and the NSS subgroup (Figure 3).

**FIGURE 4:**
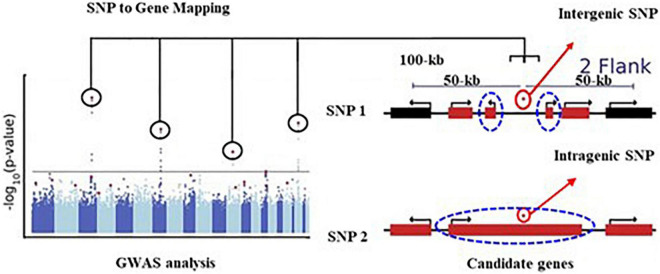
Schematic illustrations of SNP to gene mapping modified from Schoeman et al. (2016). A typical example of genotype-phenotype GWAS identified several SNPs (circled). In this study, for the intergenic SNP, DNA sequences that are located in a 100-kb window size (50 kb flanking the upstream and downstream of an SNP) the most close to the SNP was identified as regions containing candidate genes (dash blue circle). For the intragenic SNP, the genes where the SNP was located were selected as the candidate genes.

The GWAS analysis was conducted using the multi-locus random-SNP-effect mixed linear models in R package “mrMLM” (version 4.0) together with six different association methods (mrMLM, FASTmrMLM, FASTmrEMMA, ISIS EM-BLASSO, pLARmEB, and pKWmEB) to balance the false positives and false negatives. Only the SNPs that were identified by more than two methods with *p* ≤ 6.4e-7 were considered as significantly associated with the target trait. A total of 10 SNPs were identified, of which 1 SNP was identified by five, 1 SNP by four, 2 SNPs by three, and 6 SNPs by two methods (Table 1). For example, the SNP chr2.S_7040126 on chromosome 1 was co-detected by five GWAS methods. Thereafter, these 10 unique SNPs were used to identify candidate genes, of which 9 SNPs were located in the intergenic regions, and 1 was situated in the intragenic region (Table 1). Here, a 100-kb window flanking 50-kb upstream and downstream of each intergenic SNP was defined as the candidate gene selection region (as shown in the schematic presentation of Figure 4). The selection of the 100-kb window size was based on a similar GWAS population in maize for the identification of candidate genes associated with a given locus (Li et al., 2013). Two flanking genes that were most close to the SNP were selected as the candidate genes. The intragenic SNPs were referred to as those located within a given candidate gene. For example, the chr2.S_13594672 is located in the exon region of the *Zm00001d002477* gene, which was thus selected as the candidate gene.

**TABLE 1:** Significant SNPs associated with a trait of total cavity detected by muti-GWAS methods.

SNP	Biotytpe	Gene	Annotation	(Number) of methods*
chr2.S_7040126	Intergenic	*Zm00001d002164*	Glucuronosyltransferase pseudogene	(5) mrMLM, FASTmrMLM, FASTmrEMMA, pLARmEB, pKWmEB
		*Zm00001d002166*	Eukaryotic translation initiation factor 3 subunit C/EIF3C	
chr9.S_26820186	Intergenic	*Zm00001d045562*	Late embryogenesis abundant protein	(4) FASTmrMLM, pLARmEB, pKWmEB, ISIS EM-BLASSO
		*Zm00001d045563*	Dwarf plant 3, GA12 biosynthesis	
chr4.S_145983228	Intergenic	*Zm00001d051149*	Transcription factor Myb39	(3) FASTmrMLM, pKWmEB, ISIS EM-BLASSO
		*Zm00001d051151*	Uncharacterized	
chr5.S_3094291	Intergenic	*Zm00001d013005*	Ring-type E3 ubiquitin transferase	(3) mrMLM, FASTmrMLM, ISIS EM-BLASSO
		*Zm00001d013006*	Topoisomerase (ATP-hydrolyzing), Probable DNA gyrase subunit A (GyrA)	
chr9.S_12428592	Intergenic	*Zm00001d045097*	Multidrug resistance-associated protein 11	(2) FASTmrMLM, pLARmEB
		*Zm00001d045098*	Uncharacterized	
chr1.S_255324719	Intergenic	*Zm00001d033236*	Uncharacterized	(2) FASTmrMLM, pLARmEB
		*Zm00001d033237*	Uncharacterized	
chr5.S_5898418	Intergenic	*Zm00001d013173*	PHLOEM PROTEIN 2-LIKE A10	(2) pKWmEB, ISIS EM-BLASSO
		*Zm00001d013175*	Uncharacterized	
chr8.S_133443851	Intergenic	*Zm00001d010919*	Pto kinase interactor 1	(2) mrMLM, FASTmrMLM
		*Zm00001d008093*	Uncharacterized	
chr2.S_230887435	Intergenic	*Zm00001d007401*	Uncharacterized	(2) pLARmEB, ISIS EM-BLASSO
		*Zm00001d007403*	Chalcone synthase (whp)	
chr2.S_13594672	Exonic	*Zm00001d002477*	Uncharacterized	(2) FASTmrMLM, pKWmEB

*The (Number) of methods means the SNP co-detected by the number of different GWAS methods.

**FIGURE 5:**
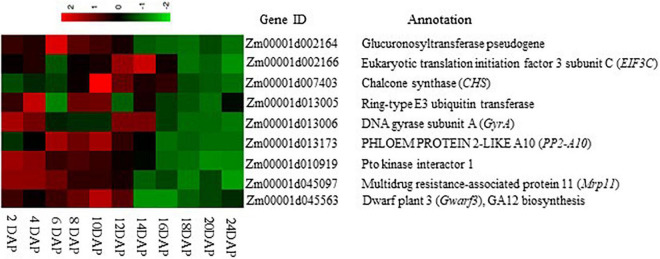
Expression heat map of the nine candidate genes linked to endosperm cavity during maize grain development. The heat map was drawn according to the FPKM value of each gene at different developmental stages of the B73 maize kernel, which was extracted from Stelpflug et al. (2016) through the qTeller platform.

In total, 19 candidate genes were identified through this exercise (Table 1). All the candidate genes were annotated according to the maize B73 reference genome (B73 RefGen_v4) available in Ensembl-Plants and NCBI Gene database. Among them, eight genes were annotated to proteins with unknown function, while the other 11 out of 19 candidate genes were annotated encoding specific proteins (Table 1). Moreover, data-mining of qTeller^4^ expression profiles revealed that 9 out of the 11 candidate genes were expressed during the EC formation (Figure 5). These include genes that encode proteins functioning as enzymes, transcription factors, and those used for gibberellin biosynthesis, as described below.

Here, candidate gene *Zm00001d001164* encodes a glucuronosyltransferase, which functions in mitochondrial electron transport transferring electrons from succinate to ubiquinone. *Zm00001d002166* encodes the eukaryotic translation initiation factor 3 subunit C (eif3c) that participates in most of the translation initiation processes. In Arabidopsis, mutation analysis of *Ateif3c1* caused embryo development defects, leading to seed abortion (Raabe et al., 2019). *Zm00001d045563* (*Dwarf3*) encodes cytochrome P450 enzyme, which functions in the early step of gibberellin synthesis (Winkler and Helentjaris, 1995). *Zm00001d013005* encodes a ring-type E3 ubiquitin transferase. It functions in the final step of the ubiquitination process (Zhou and Zeng, 2017) and is critical to the plant immunity system (Park et al., 2016). *Zm00001d013006* was annotated to encode a DNA gyrase subunit A (GyrA) of DNA topoisomerase, which is a key enzyme that controls the topological state of DNA (Champoux, 2001). DNA gyrase is the type II topoisomerases, essential for DNA replication and transcription (Champoux, 2001). Interestingly, one of the candidate genes, *Zm00001d045097* was found to encode a multidrug resistance-associated protein 11 (MRP11), which belongs to the subfamily of ATP-binding cassette transporter (ABC-transporter) family. Previous studies revealed that the MRP proteins play important roles in plant detoxification by sequestrating toxic metabolites from the cytoplasm to the large central vacuole, thereby preventing cellular damage (Klein et al., 2006). For example, in maize, ZmMrp3 was localized to tonoplasts for anthocyanin transportation (Goodman et al., 2004). *Zm00001d013173*, which was annotated as a *Phloem protein 2* (*PP2*) like gene, encodes a PHLOEM PROTEIN 2-LIKE A10 (PP2-A10). Despite little information available on *PP2-A10* in maize, its Arabidopsis ortholog *AtPP2-A10* was identified as a member of the Arabidopsis lectin family gene (Dinant et al., 2003; Naithani et al., 2021). Lectin family proteins possess at least one carbohydrate recognition domain (CRD), which enables reversible binding to carbohydrates. This is critical for maintaining sugar-related osmotic balance in the plant cell *via* endogenous lectin cycle, in which lectin binds to sugar to form a sugar-complex rendering them osmotically inert when sugar becomes surplus and release sugar for utilization when needed (Nonomura et al., 2020). *Zm00001d010919* encodes a protein kinase superfamily protein, Pto kinase interactor1 with unknown biological function, whereas *Zm00001d007403* encodes a chalcone synthase (CHS), which functions in the synthesis of maysin in maize, which is a C-glycosyl flavone found in maize silk tissue that confers resistance to corn earworm (Meyer et al., 2007).

## Discussion

In this study, we investigated the timing and position of EC formation during kernel development by using X-ray μCT scanning coupled with a histological examination followed by GWAS analyses for SNPs and candidate genes linking EC formation using the standard B73 maize inbred lines. The analyses showed that the EC was formed in the starch endosperm at 12 DAP and expanded further at 14 DAP (Figure 1). Noticeably, the histological analysis clearly demonstrated that the EC was initiated at the early stage of storage product accumulation, as supported by the observation of starch granule deposition at 10 DAP onwards, concomitant with the formation of EC (Figures 1G–J). This is consistent with previous reports that starch accumulation in maize grains starts from 8–10 DAP and lasts for about 30 days (Zhan et al., 2015).

Cellular degeneration, hence the formation of the cavity has been observed in the 12 DAP endosperm of the *shrunken1* (*sh1*) single or *sh1sus1 double* mutant, primarily due to the loss of the corresponding sucrose synthase proteins, leading to reduced UDP-glucose levels for cell wall cellulose synthesis and to a less extent for starch synthesis (Chourey et al., 1998). Related to this study, the *Shrunken2* maize mutant that lacks one of the subunits of ADP-Glc pyrophosphorylase, a key enzyme required for starch synthesis, exhibited a significant reduction of starch content, accompanied by the formation of a hollow space at the endosperm region at 16–24 DAP (Young et al., 1997). It is important to note, however, in both scenarios, no EC was observed from the respective wild-type background. This is most likely due to the EC in the WT grain being relatively small and missed from the traditional histological examinations on a one-dimensional section in those studies (Young et al., 1997; Chourey et al., 1998). Our X-ray μCT scanning thus revealed for the first time that EC was indeed formed within wild-type endosperm at 12 DAP.

The *shrunken1* (*sh1*) single or *sh1sus1 double* mutant (Chourey et al., 1998) and *shrunken2* (*sh2*) mutant (Young et al., 1997) exhibited the formation of EC due to programmed cell death (PCD) in the endosperm region. Further analysis revealed that the cellular degeneration of *shrunken1* (*sh1*) single or *sh1sus1 double* arose from a lack of cell wall synthesis precursor UDP-glucose, which affects the cell wall assembly and stability (Chourey et al., 1998), while the accumulation of ethylene, as a result of increased soluble sugars, may have trigged PCD in the endosperm region of the *shrunken2* (*sh2*) mutant (Young et al., 1997). However, the two models discussed above do not explain why EC is formed in the wild-type grain where none of the above mutations occur and the C status remains unaltered.

Our investigation on WT maize grains integrating X-ray μCT phenotyping with GWAS identified a cohort of EC-associated genes, none of which are immediately associated with PCD. Interestingly, a group of candidate genes that encode enzymes, transporters, and lectin were identified (Table 1). For example, *Zm00001d013005* encodes a ring-type E3 ubiquitin transferase. It functions at the final step of the ubiquitination process (Zhou and Zeng, 2017) and is critical to the plant immunity system (Park et al., 2016). These findings suggest a novel pathway underlying the formation of EC in the WT maize grains.

Among the group of candidate genes, *Zm00001d045097*, encoding a multidrug resistance-associated protein (*ZmMrp11*), a subfamily of the ABC transporter family, is of particular interest. Mrp11 protein has been reported to be involved in vacuolar sequestration of potentially toxic metabolites from the cytoplasm, hence preventing cellular damage (Klein et al., 2006). Interestingly, *ZmMrp11* displayed a reduced expression during the maize kernel development starting from 6–10 DAP (Figure 5; Stelpflug et al., 2016), prior to the EC formation (Figure 1), indicating the role of ZmMrp11 in the development of EC. We speculate that the downregulation of *ZmMrp11* may block the sequestration of potentially toxic compounds to the vacuole, resulting in their accumulation in the cytoplasm and consequently cell death, hence the formation of EC. Further studies are required to examine what triggers the decreased expression of *ZmMrp11* in maize endosperm and whether sustaining its expression during grain development may prevent the formation of EC.

Apart from the possible role of *ZmMrp11* in EC formation as discussed above, other candidate genes identified by GWAS could also be involved in the EC formation. For example, the glucuronosyltransferase encoded by *Zm00001d002164* may function in mitochondrial electron transport, which is critical for maintaining respiration and ATP generation. Its decreased expression from 6–10 DPA onward in maize grain (Figure 5) may compromise primary metabolism in the endosperm, contributing to cell death. To this end, it is worth noting, that the O_2_ level is generally low within bulky plant organs (Geigenberger, 2003), thus cells in those regions, such as those in the deep endosperm, might be particularly sensitive to disruption in respiration. Another candidate gene is *Zm00001d013173* encoding PP2-A10, which may function as a lectin that is essential for maintaining sugar homeostasis in the plant cell (Nonomura et al., 2020). *Zm00001d013173* exhibits a decreased expression pattern during the maize kernel development and starts to decrease at 12 DAP when EC became visible (Figures 1, 5). This suggests the *Zm00001d013173* may also be involved in the process of EC formation. In this context, we hypothesize that the reduction of *Zm00001d013173* may disrupt the sugar–lectin equilibrium, leading to more sugar release from the sugar–lectin complex, which in turn, results in the disruption of sugar homeostasis or osmotic balance, subsequently cell death and the formation EC. Indeed, an increase in sugar content and osmotic potential in the maize endosperm results in the formation of EC, as a result of the silencing of *SH1* (Zhang et al., 2020). On another hand, a previous study suggested that a strawberry homolog of Zm00001d013173, CBMFaEXP2, played a role in modifying and loosening plant cell wall structure (Nardi et al., 2015). Thus, a decrease of *Zm00001d013173* during the kernel development (Figure 5) may also affect the cell wall integrity of the endosperm, thus contributing to the formation of EC. To this end, the loss of SH1 disrupted cell wall synthesis in maize endosperm, leading to cellular degradation and the formation of a cavity between the maternal pedicel and the endosperm of the *sh1* mutant (Chourey et al., 1998).

**FIGURE 6:**
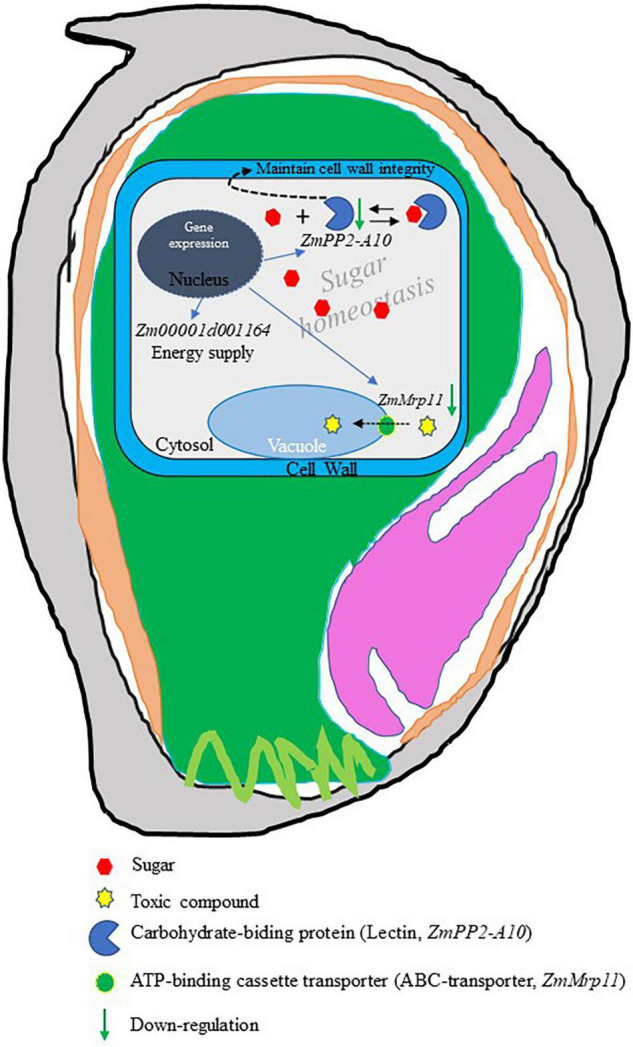
A model of endosperm cavity (EC) formation. In this model, it was proposed that programmed cell death (PCD)-trigged EC formation may be due to (i) lack of energy supply resulting from the decrease of mitochondrial electron transport transferase gene, *Zm00001d001164*. (ii) Accumulation of toxic compounds in the cytosol because downregulation of *ZmMrp11* blocked the sequestration of toxic intermediates to the vacuole. (iii) Effect on cell stability cause of loss cell wall integrity results from a decline in the expression of *ZmPP2-A10*, a possible regulator of cell wall integrity. (iv) Disruption of sugar homeostasis in the cytosol as a result of downregulation of *ZmPP2-A10*, which may function as a carbohydrate binding protein (Lectin) playing a key role in maintaining sugar homeostasis by binding the free sugar forming a sugar-lectin complex and inert sugar osmotic stress.

Based on the analyses above, we propose a model to illustrate how EC may be formed in maize grains (Figure 6). The PCD-mediated EC formation may be attributed to three factors: (i) a lack of energy, as a result of downregulation of the mitochondrial electron transport transferase gene encoding glucuronosyltransferase. This together with the lower level of O_2_ within the endosperm aggravates energy shortage, resulting in cell death; (ii) the failure of sequestration of toxic compounds to vacuoles, resulting in cytosolic toxicity and cell death, and (iii) the degradation of cell wall integrity and disruption of sugar–lectin equilibrium due to the decreased expression of the carbohydrate-binding protein (lectin) gene. It is also plausible the combination of the above three factors leads to the formation of EC. Further studies, such as genome-editing based knockout experiments, are required to determine the roles of these candidate genes in EC formation.

## Data Availability Statement

The original contributions presented in the study are included in the article/Supplementary Material, further inquiries can be directed to the corresponding author/s.

## Author Contributions

XG and Y-LR conceived the project. XG, CZ, and SL designed the experiments. SL, YZ, and JW conducted the experiments. SL, Y-LR, and XG analyzed the data and wrote the manuscript. All authors contributed to the article and approved the submitted version.

## Conflict of Interest

The authors declare that the research was conducted in the absence of any commercial or financial relationships that could be construed as a potential conflict of interest.

## Publisher’s Note

All claims expressed in this article are solely those of the authors and do not necessarily represent those of their affiliated organizations, or those of the publisher, the editors and the reviewers. Any product that may be evaluated in this article, or claim that may be made by its manufacturer, is not guaranteed or endorsed by the publisher.

## Footnotes

1
www.maizego.org/Resources.html

2
www.Plants.ensembl.org

3
www.ncbi.nlm.nih.gov/gene/

4
qteller.maizegdb.org
